# Supplementary material for: Altered feeding behavior and immune competence in paper wasps: A case of parasite manipulation?
Source: PLoS One. 2020 Dec 16;15(12):e0242486. doi: 10.1371/journal.pone.0242486 (PMC7743958; doi:10.1371/journal.pone.0242486)

**S1 File. Preference bioassays**

**S1 Table** – Mean (SE) foraging time (sec) spent by wasps on fresh buds and treated buds (withered, ethanol and hexane washed); parasitized and non-parasitized wasps on fresh buds.

| untreated vs treated buds | N | treated buds | fresh buds | parasitized wasps on fresh buds | non-parasitized wasps on fresh buds |
| --- | --- | --- | --- | --- | --- |
| trial 1: fresh and withered | 32 | 5.68 (1.22) | 19.68 (0.36) | 22.82 (1.61) | 16.13 (1.46) |
| trial 2: fresh and ethanol washed | 27 | 4.59 (0.37) | 17.15 (1.11) | 20.85 (0.84) | 13.15 (1.49) |
| trial 3: fresh and hexane washed | 24 | 4.58 (0.37) | 16.92 (1.28) | 20.38 (1.37) | 12.41 (1.48) |

**S2 Table**. Post-hoc mean-separation test (Tukey adjusted method) conducted on the significant interaction (parasite:bud condition). All combinations of factors were significant, except for no parasite, treated bud - parasite, treated bud. Results are averaged over the levels of: rearing environment, bud treatment.

| parasite-bud condition | lsmean | SE | df | lower.CL | upper.CL |
| --- | --- | --- | --- | --- | --- |
| no parasite-fresh bud | 13.99 | 0.632 | 156 | 12.39 | 15.58 |
| parasite-fresh bud | 21.36 | 0.594 | 156 | 19.86 | 22.86 |
| no parasite-treated bud | 5.06 | 0.632 | 156 | 3.47 | 6.66 |
| parasite-treated bud | 4.77 | 0.594 | 156 | 3.27 | 6.27 |

Confidence level used: 0.95 Conf-level adjustment: sidak method for 4 estimates.

| CONTRAST | estimate | SE | df | t. ratio | P value |
| --- | --- | --- | --- | --- | --- |
| no parasite, fresh bud - parasite, fresh bud | -7.372 | 0.868 | 156 | -8.497 | <.0001 |
| no parasite, fresh bud -no parasite, treated bud | 8.923 | 0.889 | 156 | 10.033 | <.0001 |
| no parasite, fresh bud - parasite, treated bud | 9.219 | 0.868 | 156 | 10.625 | <.0001 |
| parasite, fresh bud - no parasite, treated bud | 16.295 | 0.868 | 156 | 18.780 | <.0001 |
| parasite, fresh bud - parasite, treated bud | 16.591 | 0.837 | 156 | 19.815 | <.0001 |
| no parasite, treated bud - parasite, treated bud | 0.296 | 0.868 | 156 | 0.341 | 0.9863 |

P value adjustment: Tukey method for comparing a family of 4 estimates

**S1 Fig. Interaction plot.**

Interaction plot is performed to visualize the significant interaction effect between parasite and bud condition. The bud condition was set as trace variable and the time (sec) as response variable. Parite was reported on x-axis and the mean of time for each group was reported on y-axis.


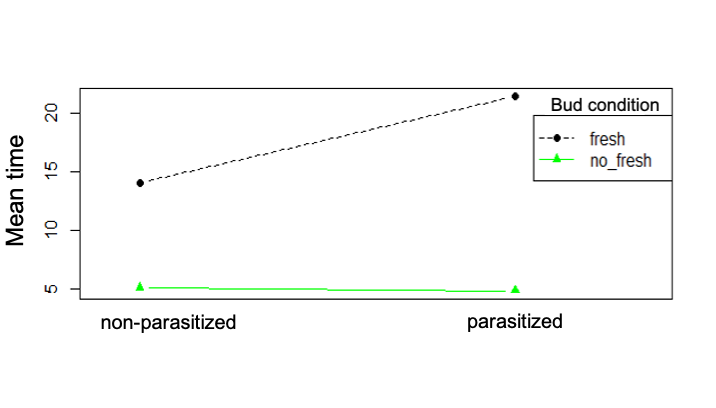

Supplement: S1 File — (DOCX) [file pone.0242486.s001.docx]
