# Supplementary material for: Altered feeding behavior and immune competence in paper wasps: A case of parasite manipulation?
Source: PLoS One. 2020 Dec 16;15(12):e0242486. doi: 10.1371/journal.pone.0242486 (PMC7743958; doi:10.1371/journal.pone.0242486)
Supplement: S2 File — (DOCX) [file pone.0242486.s002.docx]

**S2 File. Verbascoside and chromatographic profiles at different wavelengths**

Verbascoside, also known as acteoside (Birkofer et al. 1968; Chen et al. 2012) or kusaginin (Sakurai and Kato 1983), is a water-soluble phenolic compound belonging to the phenylethanoid glycoside family, widely distributed in the plant kingdom. The HPLC-DAD analysis of the extract administrered to wasps confirmed the proportion of 10% ofverbascoside. Briefly, 3mg of dry extract were dissolved in 10 mL of MeOH:H_2_O 80:20 v/v. The final concentration of verbascoside was 0.0333 mg/mL, corresponding to 10.54% w/w of verbascoside in the extract.The mass of verbascoside was measured and the accuracy was checked by analyzing verbascoside standard in the same conditions of *C. radicans* samples.

**S2 Fig. Mass spectra of verbascoside**

**
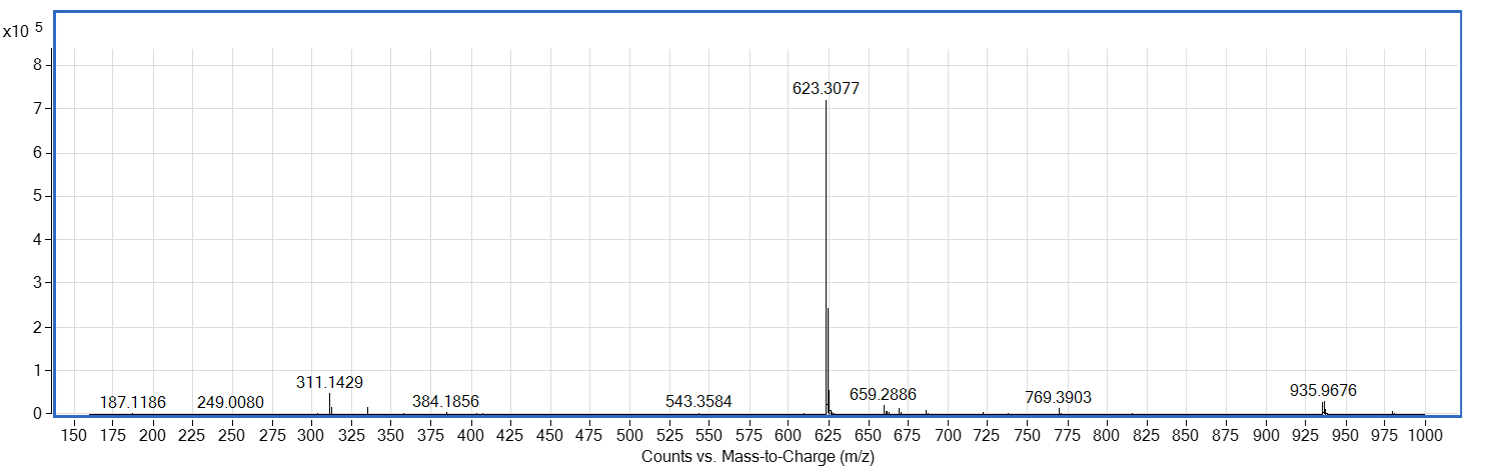
**

**Chromatographic profiles at different wavelenghts**

The zoom of the chromatographic profiles at 280 nm, 260 nm and 330 nm of an ethanol extract from calix tissue of *C. radica*ns rich of EFNs allowed highlighting the presence of some minor secondary metabolites detected in a very low amount, as confirmed by the maximum intensity of the signals at the different wavelenghts.

Figure S3 shows the chromatogram as such and a 100× zoom in the range 7-19 minutes. As it clearly appears, verbascoside is largely the main compound. Among the minor peaks, the biggest two (retention time about 12.0 and 12.6 minutes) have been identified as verbascoside related compounds, according to the very similar UV-vis spectra. The mass spectra indicate a molecular weight of 638 Da and 668 Da, which allowed tentatively identifying them as ethyl-β-OH-acteoside (668 Da) and eukovoside (638 Da). The amount of these two peaks is below 5% of the verbascoside content.

MS analysis also allowed identifying further compounds present in trace amount as the verbascoside related molecules β-OH-acteoside isomer 1 and β-OH-acteoside isomer 2, and the flavonol rutin.

**S3 Fig. Chromatographic profiles, its 100-fold zoom and chemical structure of tentatively identified molecules**

References

Birkofer, L., Kaiser, C., Thomas, U. (1968). Acteoside and neoacteoside: Zuckerester aus *Syringa vulgaris*. Naturforschung 236, 1051-1058.

Chen, C. H., Lin, Y. S., Chien, M. Y., Hou, W. C., Hu. M. L. (2012) Antioxidant and antihypertensive activities of acteoside and its analogs. Botanical Studies 53, 421-429.

Sakurai, A., Kato, T. (1983). A new glycoside, kusaginin isolated from Clerodendron trichotomum. *Bulletin of the Chemical Society of Japan*, 56, 1573-1574.
